# Supplementary material for: The FKBP51 Inhibitor SAFit2 Restores the Pain-Relieving C16 Dihydroceramide after Nerve Injury
Source: Int J Mol Sci. 2022 Nov 17;23(22):14274. doi: 10.3390/ijms232214274 (PMC9695264; doi:10.3390/ijms232214274)
Supplement: Supplementary file 1 [file ijms-23-14274-s001.zip › ijms-2023438-supplementary.pdf]

Supplemental Information

The FKBP51 inhibitor SAFit2 restores the pain relieving C16 dihydroceramide after nerve injury

Saskia Wedel[1], Lisa Hahnefeld[1,5], Mohamad Wessam Alnouri[2], Stefan Offermanns[2,3], Felix Hausch[4], Gerd Geisslinger[1,5] and Marco Sisignano[1,5*]

[1] Institute of Clinical Pharmacology, *pharmazentrum frankfurt*/ZAFES, University Hospital, Goethe-University, 60590 Frankfurt am Main, Germany

[2] Max Planck Institute for Heart and Lung Research, 61231 Bad Nauheim, Germany

[3] Center for Molecular Medicine, Goethe-University Frankfurt, 60590 Frankfurt am Main, Germany

[4] Department of Biochemistry, Technical University Darmstadt, 64287 Darmstadt, Germany

[5] Fraunhofer Institute for Translational Medicine and Pharmacology ITMP, and Fraunhofer Cluster of Excellence for Immune Mediated Diseases CIMD, 60596 Frankfurt am Main, Germany

*Address correspondence to: Dr. Marco Sisignano, *pharmazentrum frankfurt*/ZAFES, Institute of Clinical Pharmacology, University Hospital, Goethe-University, D-60590 Frankfurt am Main, Germany, Phone: +49 (0)69-6301-7819, E-mail: Marco.Sisignano@med.uni-frankfurt.de

Supplementary Figures: 8

Supplementary Tables: 3

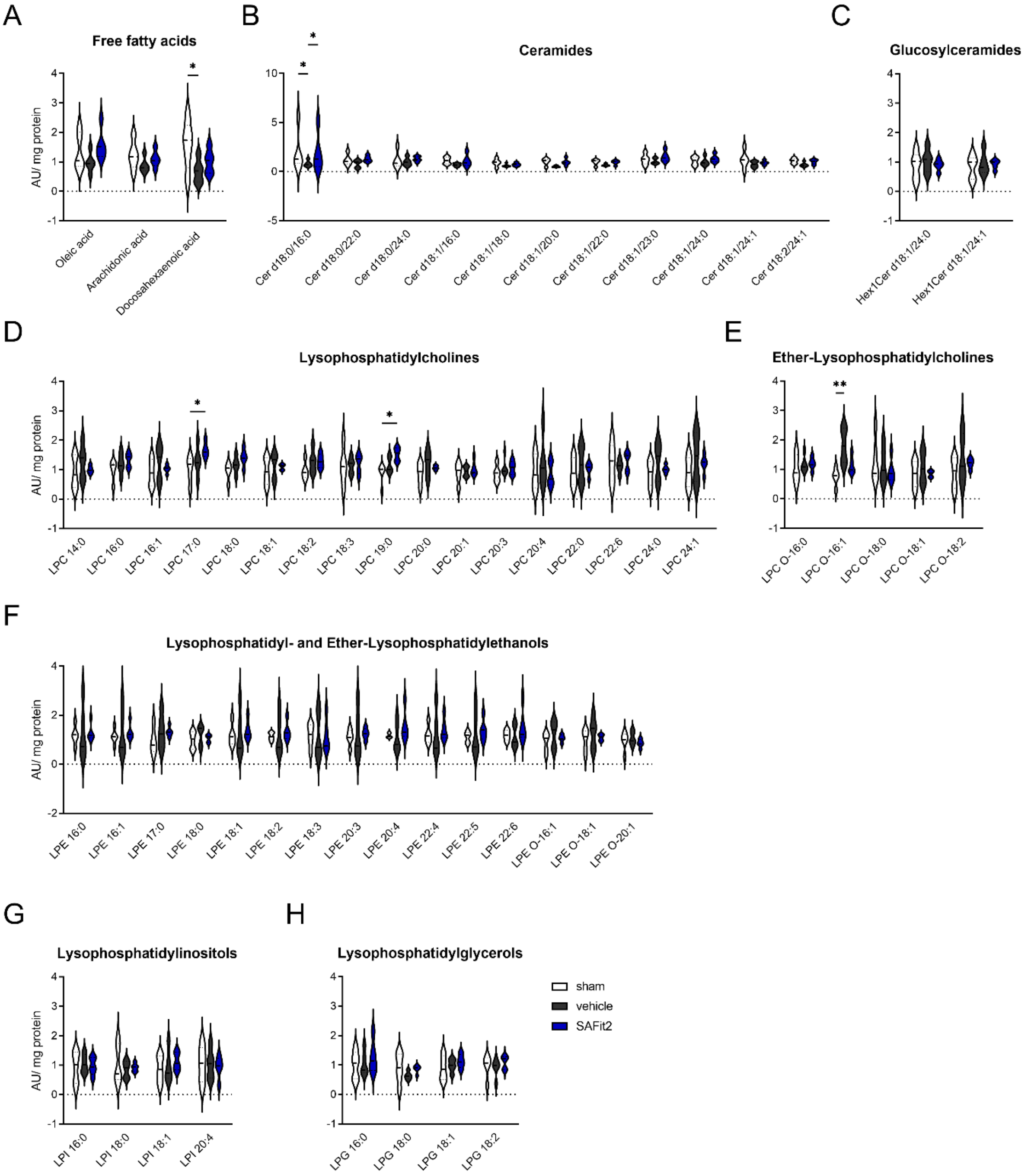

Figure S1: Normalized lipid concentrations of free fatty acids, ceramides and lysophospholipids in lumbar DRGs 21 days after SNI. Mice underwent SNI surgery and were treated with either vehicle or 10 mg/kg SAFit2 from day five to ten after the surgery. After 21 days, the lumbar DRGs were isolated from ipsilateral and contralateral sides, lysed and lipids were measured in an untargeted LC-HRMS screening. The distribution of normalized lipids is displayed treatment-wise for each lipid, which are further grouped in classes: **(A)** fatty acids **(B)** ceramides, **(C)** glucosylceramides, **(D)** lysophohsphatidylcholines, **(E)** ether-

lysophosphatidylcholines, **(F)** lysophosphatidyl- and ether-lysophosphatidylethanols, **(G)** lysophosphatidylinositols, **(H)** lysophosphatidylglycerols. * $p < 0.05$ two-way ANOVA with Tukey´s post hoc test. Abbreviations: DRGs: dorsal root ganglia, SAFit2: selective antagonist of FKBP51 by induced fit 2, SNI: spared nerve injury, LC-HRMS: liquid chromatography-high-resolution mass spectrometry

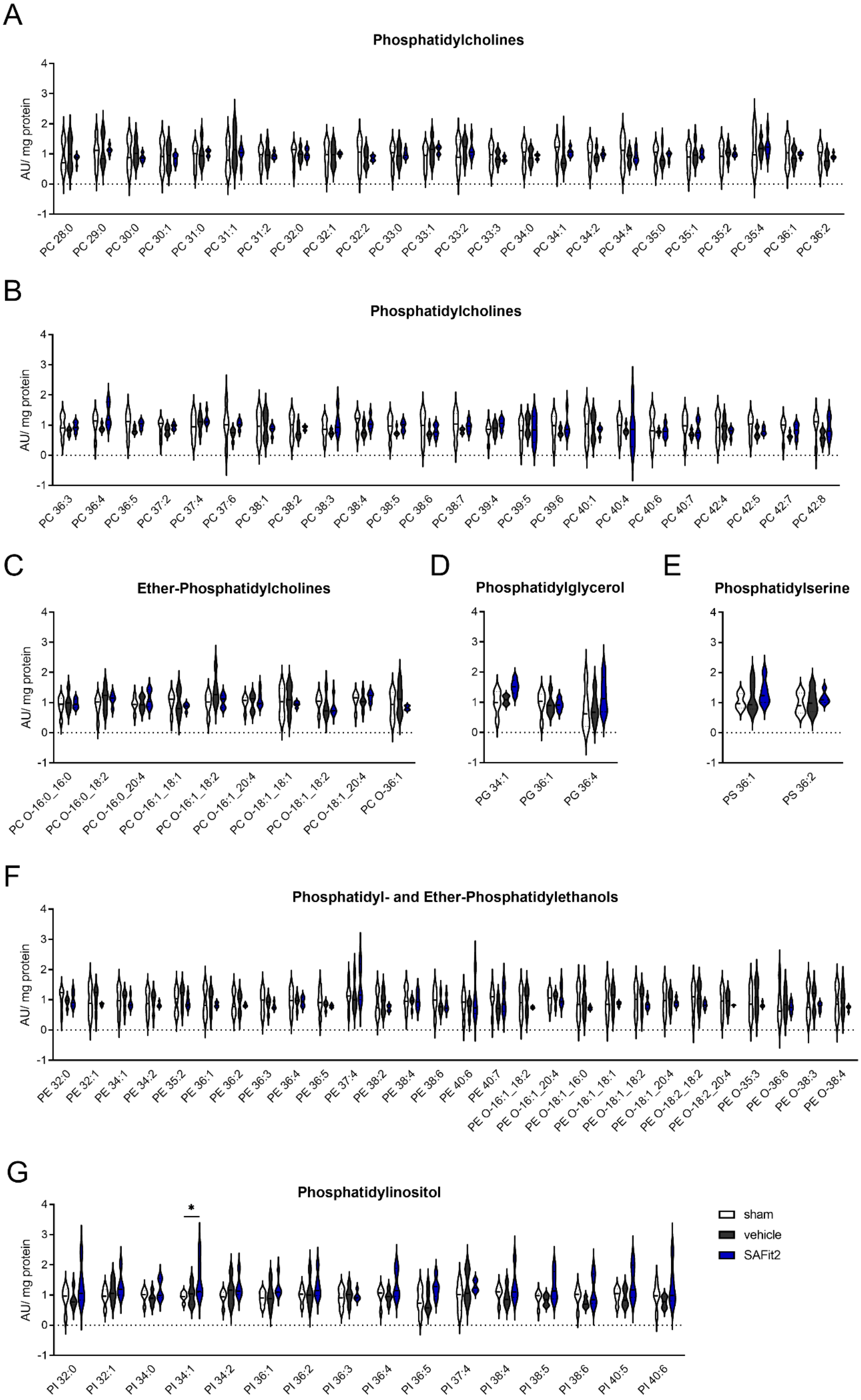
A
Phosphatidylcholines
AU/ mg protein
4
3
2
1
0
-1
PC 28:0
PC 29:0
PC 30:0
PC 30:1
PC 31:0
PC 31:1
PC 31:2
PC 32:0
PC 32:1
PC 32:2
PC 33:0
PC 33:1
PC 33:2
PC 33:3
PC 34:0
PC 34:1
PC 34:2
PC 34:4
PC 35:0
PC 35:1
PC 35:2
PC 35:4
PC 36:1
PC 36:2
B
Phosphatidylcholines
AU/ mg protein
4
3
2
1
0
-1
PC 36:3
PC 36:4
PC 36:5
PC 37:2
PC 37:4
PC 37:6
PC 38:1
PC 38:2
PC 38:3
PC 38:4
PC 38:5
PC 38:6
PC 38:7
PC 39:4
PC 39:5
PC 39:6
PC 40:1
PC 40:4
PC 40:6
PC 40:7
PC 42:4
PC 42:5
PC 42:7
PC 42:8
C
Ether-Phosphatidylcholines
AU/ mg protein
4
3
2
1
0
-1
PC O-16:0_16:0
PC O-16:0_18:2
PC O-16:0_20:4
PC O-16:1_18:1
PC O-16:1_18:2
PC O-16:1_20:4
PC O-18:1_18:1
PC O-18:1_18:2
PC O-18:1_20:4
PC O-36:1
D
Phosphatidylglycerol
4
3
2
1
0
-1
PG 34:1
PG 36:1
PG 36:4
E
Phosphatidylserine
4
3
2
1
0
-1
PS 36:1
PS 36:2
F
Phosphatidyl- and Ether-Phosphatidylethanols
AU/ mg protein
4
3
2
1
0
-1
PE 32:0
PE 32:1
PE 34:1
PE 34:2
PE 35:2
PE 36:1
PE 36:2
PE 36:3
PE 36:4
PE 36:5
PE 37:4
PE 38:2
PE 38:4
PE 38:6
PE 40:6
PE 40:7
PE O-16:1_18:2
PE O-16:1_20:4
PE O-18:1_16:0
PE O-18:1_18:1
PE O-18:1_18:2
PE O-18:1_20:4
PE O-18:2_18:2
PE O-18:2_20:4
PE O-35:3
PE O-36:6
PE O-38:3
PE O-38:4
G
Phosphatidylinositol
AU/ mg protein
4
3
2
1
0
-1
*
PI 32:0
PI 32:1
PI 34:0
PI 34:1
PI 34:2
PI 36:1
PI 36:2
PI 36:3
PI 36:4
PI 36:5
PI 37:4
PI 38:4
PI 38:5
PI 38:6
PI 40:5
PI 40:6
sham
vehicle
SAFit2

Figure S2: Normalized lipid concentrations of phosphatidylcholines, -glycerols, -serines, -ethanols and -inositols in lumbar DRGs 21 days after SNI. Mice underwent SNI surgery and were treated with either vehicle or 10 mg/kg SAFit2 from day five to ten after the surgery. After 21 days, the lumbar DRGs were isolated from ipsilateral and contralateral sides, lysed and lipids were measured in an untargeted LC-HRMS screen. The distribution of normalized lipids is displayed treatment-wise for each lipid, which are further grouped in classes: **(A, B)** phosphatidylcolines, **(C)** ether-phosphatidylcholines, **(D)** phosphatidylglycerols, **(E)** phosphatidylserines, **(F)** phosphatidyl- and ether-phosphatidylethanols, **(G)** phosphatidylinositols. * $p < 0.05$ two-way ANOVA with Tukey´s post hoc test. Abbreviations: DRGs: dorsal root ganglia, SAFit2: selective antagonist of FKBP51 by induced fit 2, SNI: spared nerve injury, LC-HRMS: liquid chromatography-high-resolution mass spectrometry

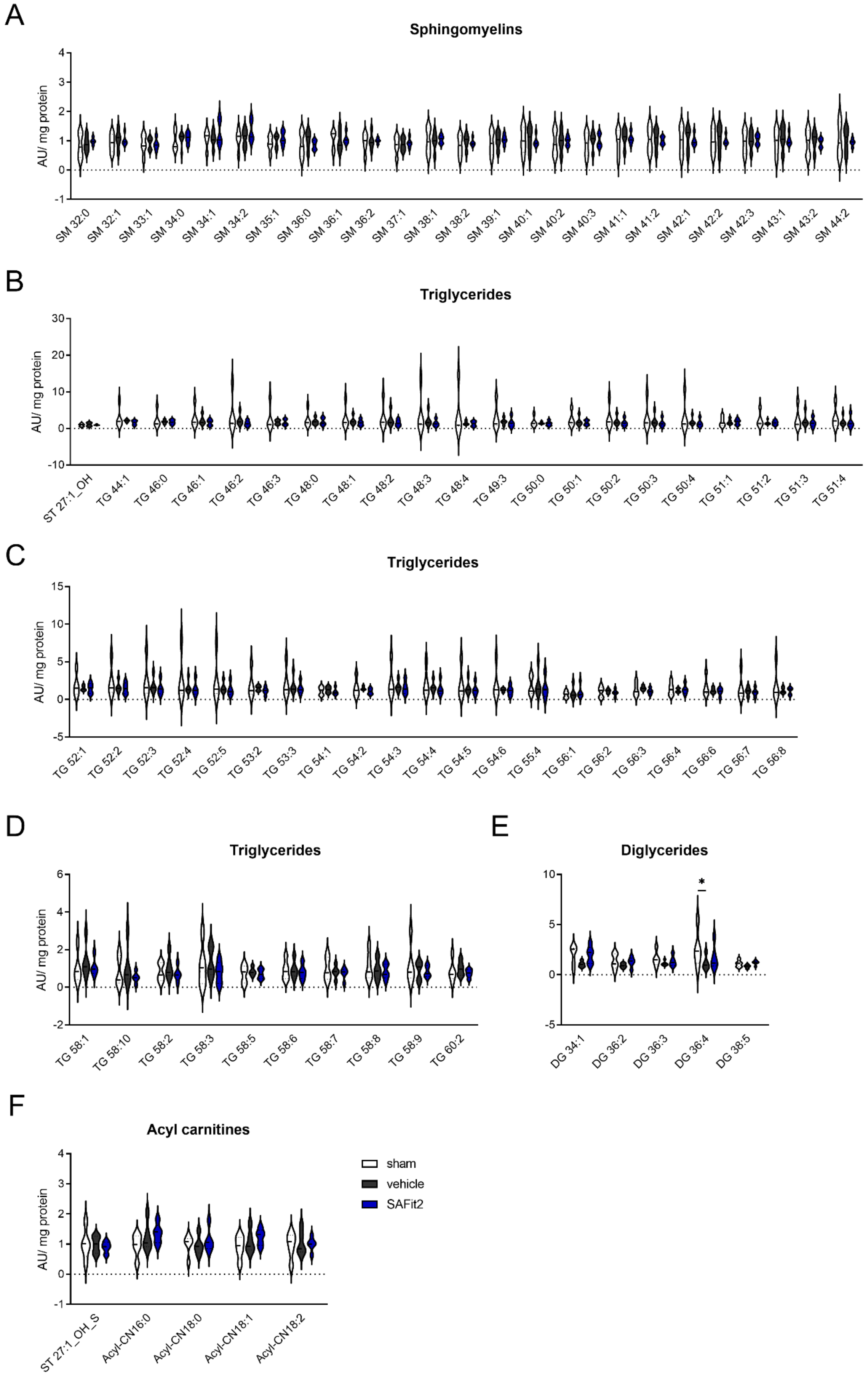
A
Sphingomyelins
AU/ mg protein
SM 32:0
SM 32:1
SM 33:1
SM 34:0
SM 34:1
SM 34:2
SM 35:1
SM 36:0
SM 36:1
SM 36:2
SM 37:1
SM 38:1
SM 38:2
SM 39:1
SM 40:1
SM 40:2
SM 40:3
SM 41:1
SM 41:2
SM 42:1
SM 42:2
SM 42:3
SM 43:1
SM 43:2
SM 44:2
B
Triglycerides
AU/ mg protein
ST 27:1_OH
TG 44:1
TG 46:0
TG 46:1
TG 46:2
TG 46:3
TG 48:0
TG 48:1
TG 48:2
TG 48:3
TG 48:4
TG 49:3
TG 50:0
TG 50:1
TG 50:2
TG 50:3
TG 50:4
TG 51:1
TG 51:2
TG 51:3
TG 51:4
C
Triglycerides
AU/ mg protein
TG 52:1
TG 52:2
TG 52:3
TG 52:4
TG 52:5
TG 53:2
TG 53:3
TG 54:1
TG 54:2
TG 54:3
TG 54:4
TG 54:5
TG 54:6
TG 55:4
TG 56:1
TG 56:2
TG 56:3
TG 56:4
TG 56:6
TG 56:7
TG 56:8
D
Triglycerides
AU/ mg protein
TG 58:1
TG 58:10
TG 58:2
TG 58:3
TG 58:5
TG 58:6
TG 58:7
TG 58:8
TG 58:9
TG 60:2
E
Diglycerides
*
DG 34:1
DG 36:2
DG 36:3
DG 36:4
DG 38:5
F
Acyl carnitines
AU/ mg protein
ST 27:1_OH_S
Acyl-CN16:0
Acyl-CN18:0
Acyl-CN18:1
Acyl-CN18:2
sham
vehicle
SAFit2

Figure S3: Normalized lipid concentrations of sphingomyelins, triglycerides, diglycerides and acyl carnitines in lumbar DRGs 21 days after SNI. Mice underwent SNI surgery and were treated with either vehicle or 10 mg/kg SAFit2 from day five to ten after the surgery. After 21 days, the lumbar DRGs were isolated from ipsilateral and contralateral sides, lysed and lipids were measured in an untargeted LC-HRMS screen. The distribution of normalized lipids is displayed treatment-wise for each lipid, which are further grouped in classes: **(A)** sphingomyelins, **(B-D)** triglycerides, **(E)** diglycerides, **(F)** acyl carnitines. * $p < 0.05$ two-way ANOVA with Tukey´s post hoc test. Abbreviations: DRGs: dorsal root ganglia, SAFit2: selective antagonist of FKBP51 by induced fit 2, SNI: spared nerve injury, LC-HRMS: liquid chromatography-high-resolution mass spectrometry

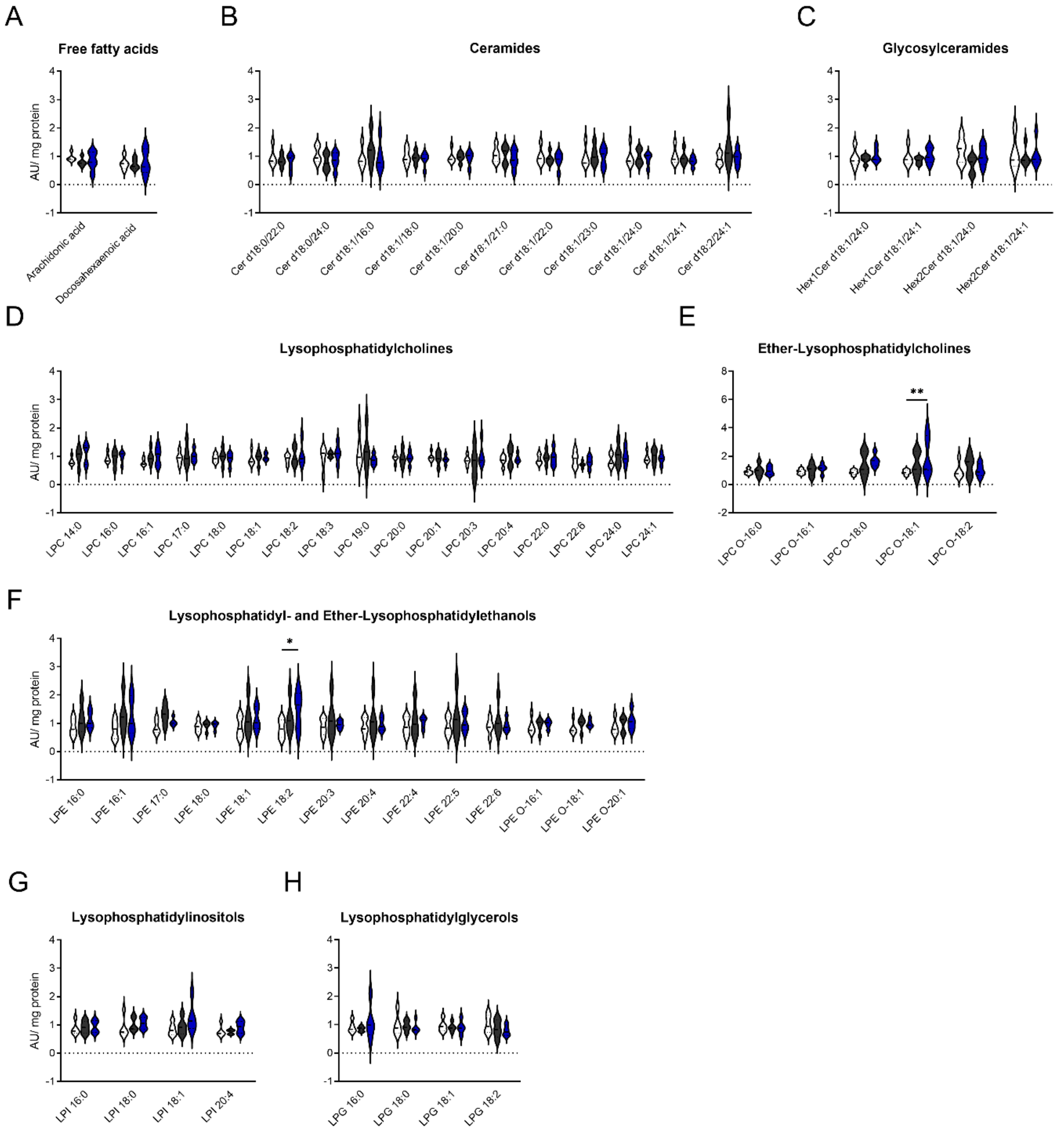

Figure S4: Normalized lipid concentrations of free fatty acids, ceramides and lysophospholipids in spinal cord 21 days after SNI. Mice underwent SNI surgery and were treated with either vehicle or 10 mg/kg SAFit2 from day five to ten after the surgery. After 21 days, the spinal cord was isolated from ipsilateral and contralateral sides, lysed and lipids were measured in an untargeted LC-HRMS screen. The distribution of normalized lipids is displayed treatment-wise for each lipid, which are grouped in classes: **(A)** fatty acids **(B)** ceramides, **(C)** glycosylceramides, **(D)** lysophohsphatidylcholines, **(E)** ether-lysophosphatidylcholines, **(F)** lysophosphatidyl-

and ether-lysophosphatidylethanols, **(G)** lysophosphatidylinositols, **(H)** lysophosphatidylglycerols. * $p < 0.05$ two-way ANOVA with Tukey´s post hoc test. Abbreviations: SC: spinal cord, SAFit2: selective antagonist of FKBP51 by induced fit 2, SNI: spared nerve injury, LC-HRMS: liquid chromatography-high-resolution mass spectrometry

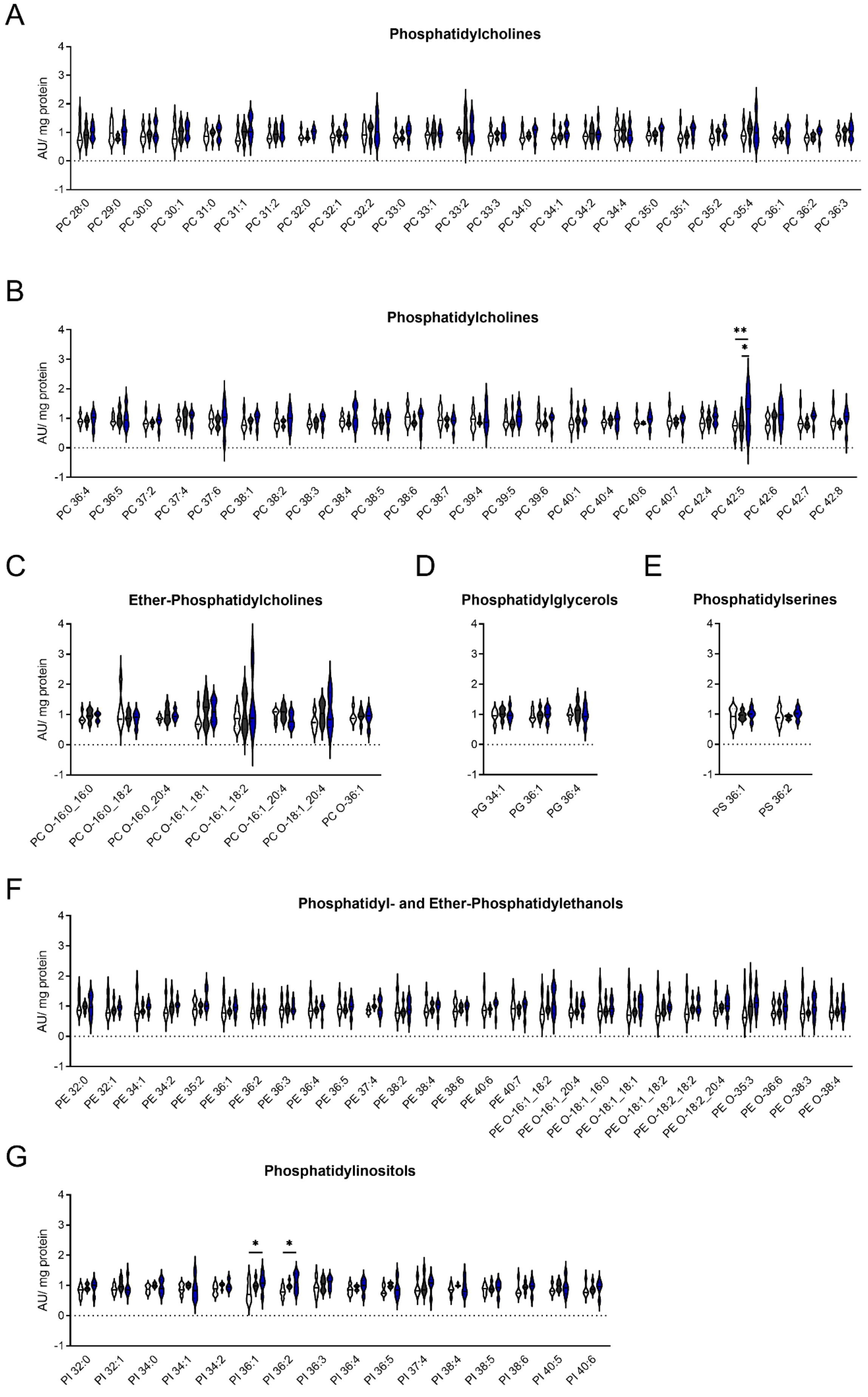
A
Phosphatidylcholines
AU/ mg protein
4
3
2
1
0
-1
PC 28:0
PC 29:0
PC 30:0
PC 30:1
PC 31:0
PC 31:1
PC 31:2
PC 32:0
PC 32:1
PC 32:2
PC 33:0
PC 33:1
PC 33:2
PC 33:3
PC 34:0
PC 34:1
PC 34:2
PC 34:4
PC 35:0
PC 35:1
PC 35:2
PC 35:4
PC 36:1
PC 36:2
PC 36:3
B
Phosphatidylcholines
AU/ mg protein
**
*
PC 36:4
PC 36:5
PC 37:2
PC 37:4
PC 37:6
PC 38:1
PC 38:2
PC 38:3
PC 38:4
PC 38:5
PC 38:6
PC 38:7
PC 39:4
PC 39:5
PC 39:6
PC 40:1
PC 40:4
PC 40:6
PC 40:7
PC 42:4
PC 42:5
PC 42:6
PC 42:7
PC 42:8
C
Ether-Phosphatidylcholines
AU/ mg protein
PC O-16:0_16:0
PC O-16:0_18:2
PC O-16:0_20:4
PC O-16:1_18:1
PC O-16:1_18:2
PC O-16:1_20:4
PC O-18:1_20:4
PC O-36:1
D
Phosphatidylglycerols
PG 34:1
PG 36:1
PG 36:4
E
Phosphatidylserines
PS 36:1
PS 36:2
F
Phosphatidyl- and Ether-Phosphatidylethanols
AU/ mg protein
PE 32:0
PE 32:1
PE 34:1
PE 34:2
PE 35:2
PE 36:1
PE 36:2
PE 36:3
PE 36:4
PE 36:5
PE 37:4
PE 38:2
PE 38:4
PE 38:6
PE 40:6
PE 40:7
PE O-16:1_18:2
PE O-16:1_20:4
PE O-18:1_16:0
PE O-18:1_18:1
PE O-18:1_18:2
PE O-18:2_18:2
PE O-18:2_20:4
PE O-35:3
PE O-36:6
PE O-38:3
PE O-38:4
G
Phosphatidylinositols
AU/ mg protein
*
*
PI 32:0
PI 32:1
PI 34:0
PI 34:1
PI 34:2
PI 36:1
PI 36:2
PI 36:3
PI 36:4
PI 36:5
PI 37:4
PI 38:4
PI 38:5
PI 38:6
PI 40:5
PI 40:6

Figure S5: Normalized lipid concentrations of phosphatidylcholines, -glycerols, -serines, -ethanols and -inositols in spinal cord 21 days after SNI. Mice underwent SNI surgery and were treated with either vehicle or 10 mg/kg SAFit2 from day five to ten after the surgery. After 21 days, the spinal cord was isolated from ipsilateral and contralateral sides, lysed and lipids were measured in an untargeted LC-HRMS screen. The distribution of normalized lipids is displayed treatment-wise for each lipid, which are grouped in classes: **(A, B)** phosphatidylcholines, **(C)** ether-phosphatidylcholines, **(D)** phosphatidylglycerols, **(E)** phosphatidylserines, **(F)** phosphatidyl- and ether-phosphatidylethanols, **(G)** phosphatidylinositols. * $p < 0.05$ two-way ANOVA with Tukey´s post hoc test. Abbreviations: SC: spinal cord, SAFit2: selective antagonist of FKBP51 by induced fit 2, SNI: spared nerve injury, LC-HRMS: liquid chromatography-high-resolution mass spectrometry

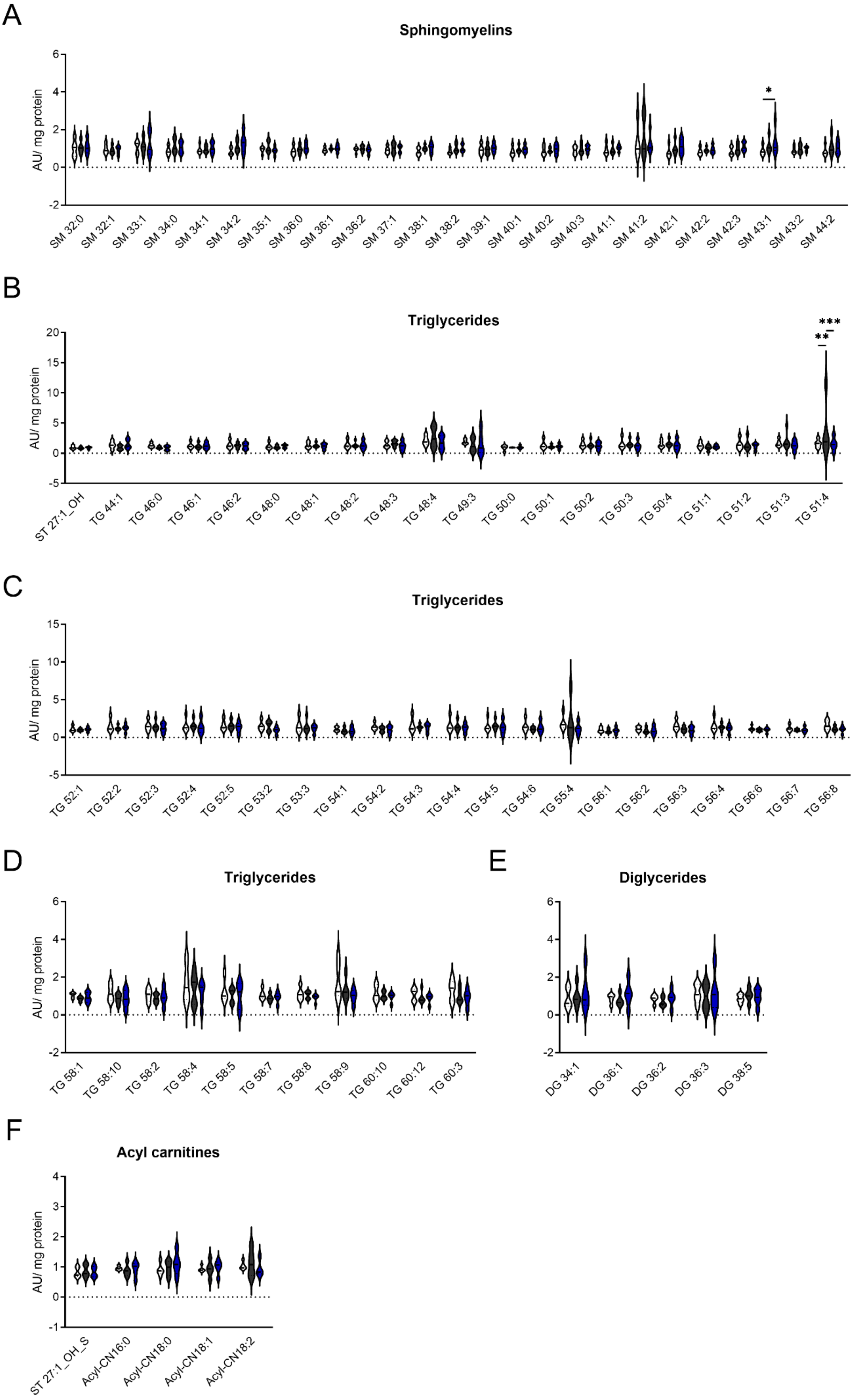
A
Sphingomyelins
AU/ mg protein
6
4
2
0
-2
*
SM 32:0
SM 32:1
SM 33:1
SM 34:0
SM 34:1
SM 34:2
SM 35:1
SM 36:0
SM 36:1
SM 36:2
SM 37:1
SM 38:1
SM 38:2
SM 39:1
SM 40:1
SM 40:2
SM 40:3
SM 41:1
SM 41:2
SM 42:1
SM 42:2
SM 42:3
SM 43:1
SM 43:2
SM 44:2
B
Triglycerides
AU/ mg protein
20
15
10
5
0
-5
***
**
ST 27:1_OH
TG 44:1
TG 46:0
TG 46:1
TG 46:2
TG 48:0
TG 48:1
TG 48:2
TG 48:3
TG 48:4
TG 49:3
TG 50:0
TG 50:1
TG 50:2
TG 50:3
TG 50:4
TG 51:1
TG 51:2
TG 51:3
TG 51:4
C
Triglycerides
AU/ mg protein
15
10
5
0
-5
TG 52:1
TG 52:2
TG 52:3
TG 52:4
TG 52:5
TG 53:2
TG 53:3
TG 54:1
TG 54:2
TG 54:3
TG 54:4
TG 54:5
TG 54:6
TG 55:4
TG 56:1
TG 56:2
TG 56:3
TG 56:4
TG 56:6
TG 56:7
TG 56:8
D
Triglycerides
AU/ mg protein
6
4
2
0
-2
TG 58:1
TG 58:10
TG 58:2
TG 58:4
TG 58:5
TG 58:7
TG 58:8
TG 58:9
TG 60:10
TG 60:12
TG 60:3
E
Diglycerides
6
4
2
0
-2
DG 34:1
DG 36:1
DG 36:2
DG 36:3
DG 38:5
F
Acyl carnitines
AU/ mg protein
4
3
2
1
0
-1
ST 27:1_OH_S
Acyl-CN16:0
Acyl-CN18:0
Acyl-CN18:1
Acyl-CN18:2

Figure S6: Normalized lipid concentrations of sphingomyelins, triglycerides, diglycerides and acyl cyanides in spinal cord after 21 days after SNI. Mice underwent SNI surgery and were treated with either vehicle or 10 mg/kg SAFit2 from day five to ten after the surgery. After 21 days, the spinal cord was isolated from ipsilateral and contralateral sides, lysed and lipids were measured in an untargeted LC-HRMS screen. The distribution of normalized lipids is displayed treatment-wise for each lipid, which are grouped in classes: **(A)** sphingomyelins, **(B-D)** triglycerides, **(E)** diglycerides, **(F)** acyl carnitines. * $p < 0.05$ two-way ANOVA with Tukey´s post hoc test. Abbreviations: SC: spinal cord, SAFit2: selective antagonist of FKBP51 by induced fit 2, SNI: spared nerve injury, LC-HRMS: liquid chromatography-high-resolution mass spectrometry

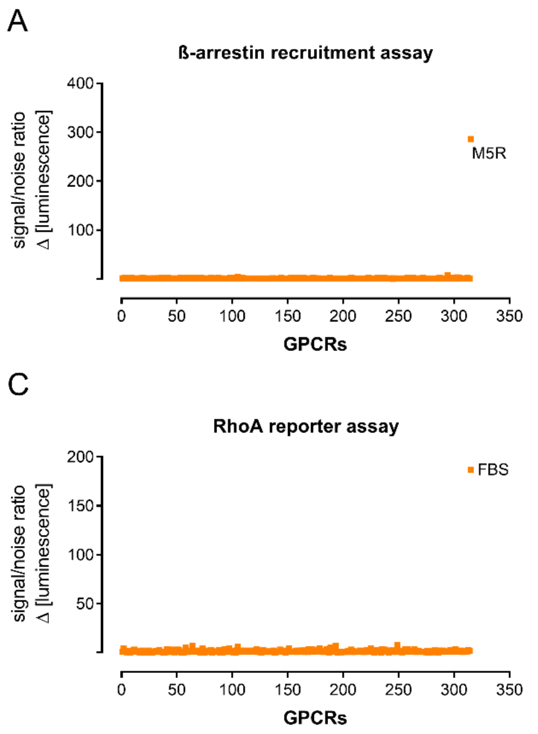

Figure S7: Results of a GPCR screen using C16 dihydroceramide as a potential interaction partner. To screen a library of 314 GPCRs in search for a potential receptor activated by the C16 dihydroceramide, a β-arrestin recruitment assay **(A)**, a cAMP assay **(B)**, and a RhoA reporter assay **(C)** were performed. The C16 was added at a final concentration of 30 µM **(A)** or 15 µM **(B,C)**. As positive controls 100 µM carbachol and the muscarinic M5 receptor **(A)**, 10 nM GLP-1 and the GLP-1 receptor **(B)**, and FBS **(C)** were used. For details see method section. The GPCRs analyzed are listed in table S1.

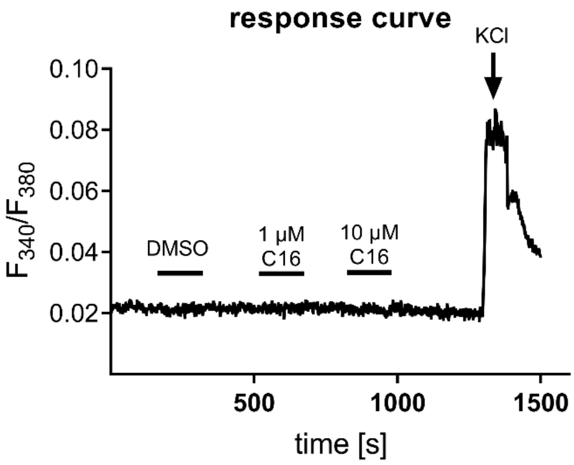

Figure S8: Representative response curve of calcium fluxes in C16 dihydroceramide treated sensory neurons. The primary cells were treated with 1 and 10 µM C16 dihydroceramide and the corresponding solvent control (DMSO) followed by a KCl (50 mM) treatment as a positive control.

Table S1: Internal standards and their concentrations in the working solutions for the lipid screening (all from Avanti Polar Lipids, Alabaster, AL, USA)

Analyte	Concentration (µg/mL)
Arachidonic acid-d8	0.1
CE 18:1-d7	5
Cer d18:1/16:0-d7	0.02
Cholesterol-d7	7.5
DG 15:0/18:1-d7	0.3
LacCer d18:1/17:0	0.06
LPC 18:1-d7	0.3
LPC O-16:0-d4	0.02
LPE 18:1-d7	0.02
LPG 17:1	0.02
LPI 17:1	0.02
PC 15:0/18:1-d7	2
PC O-18:0/18:1-d9	0.2
PE 15:0/18:1-d7	0.1
PG 15:0/18:1-d7	0.1
PI 15:0/18:1-d7	0.1
PS 15:0/18:1-d7	0.025
SM d18:1/18:1-d9	0.4
TG 14:0/16:1/14:0-d5	0.6
TG 15:0/18:1-d7/15:0	0.6
TG 20:0/20:1/20:0-d5	0.6

Table S2: LC-gradient

Time (min)	Sol. A (%)	Sol. B (%)
0.00	75.0	25.0
0.30	75.0	25.0
1.50	20.0	80.0
11.00	0.0	100.0
12.00	0.0	100.0
12.50	75.0	25.0
14.00	75.0	25.0

Instrumentation

Mass spectrometer	Orbitrap Exploris 480 (Thermo Fisher Scientific, Dreiech, Germany)
	HESI-source
HPLC	Vanquish Horizon (Thermo Fisher Scientific, Dreiech, Germany)
	Vanquish Compartment H
	Vanquish Split Sampler HT
	Vanquish Pump H
LC-column	Zorbax RRHD Eclipse Plus C8 1.8 µm 50 x 2.1 mm ID (Agilent, Waldbronn, Germany), heated at 40 °C, flow rate 300 µL/min
	Precolumn: ZORBAX Eclipse Plus C8, 2.1 mm, 1.8 µm (Agilent, Waldbronn, Germany)
Solvent A	Water + 0.1% formic acid + 10 mM ammonium formate
Solvent B	Acetonitrile:isopropanole 2:3 (v/v) + 0.1% formic acid
Injection volume	2 µL (positive ion mode), 5 µL (negative ion mode)

Table S3: List of GPCRs which were screened for potential activation by the C16 dihydroceramide

n	description	n	description	n	description	n	description	n	description	n	description
1	ADCYAP1R1	51	CX3C1	101	GPR119	151	GPR4	201	HTR1F	301	TA1
2	ADORA1	52	CXCR1	102	GPR12	152	GPR44	202	HTR2A	302	TAAR2
3	ADORA2A	53	CXCR2	103	GPR120	153	GPR45	203	HTR2B	303	TAAR5
4	ADORA2B	54	CXCR3	104	GPR123	154	GPR50	204	HTR2C	304	TAAR6
5	ADORA3	55	CXCR4	105	GPR124	155	GPR52	205	HTR4	305	TAAR8
6	ADRA1A	56	CXCR5	106	GPR125	156	GPR55	206	HTR5	306	TAAR9
7	ADRA1B	57	CXCR6	107	GPR126	157	GPR56	207	HTR6	307	TACR1
8	ADRA1D	58	CXCR7	108	GPR132	158	GPR6	208	HTR7	308	TACR2
9	ADRA2A	59	CYSLTR1	109	GPR133	159	GPR61	209	KISSPEPTIN	309	TACR3
10	ADRA2B	60	CYSLTR2	110	GPR135	160	GPR62	210	LHCGR	310	TBXA2R
11	ADRA2C	61	DRD1	111	GPR141	161	GPR63	211	LPAR1	311	TSHR
12	ADRB1	62	DRD2	112	GPR142	162	GPR64	212	LPAR2	312	UTS2R
13	ADRB2	63	DRD3	113	GPR143	163	GPR65	213	LPAR4	313	VIPR1
14	ADRB3	64	DRD4	114	GPR144	164	GPR68	214	LPAR5	314	VIPR2
15	AGTR1	65	DRD5	115	GPR146	165	GPR75	215	LPAR6		
16	AGTR2	66	EDNRA	116	GPR148	166	GPR77	216	LTB4R		
17	APJ	67	EDNRB	117	GPR149	167	GPR78	217	LTB4R2B		
18	AVPR1A	68	ELTD1	118	GPR15	168	GPR82	218	MAS1		
19	AVPR1B	69	F2R	119	GPR150	169	GPR83	219	MAS1L		
20	AVPR2	70	F2RL1	120	GPR151	170	GPR84	220	MC1R		
21	BB3	71	F2RL2	121	GPR152	171	GPR85	221	MC2R		
22	BDKRB1	72	F2RL3	122	GPR153	172	GPR87	222	MC3R		
23	BDKRB2	73	FFA1 (GPR40)	123	GPR156	173	GPR88	223	MC4R		
24	C3AR1	74	FFA2 (GPR43)	124	GPR157	174	GPR97	224	MC5R		
25	C5A	75	FFA3 (GPR41)	125	GPR158	175	GPRC5A	225	MCHR1		
26	CALCRb	76	FPR1	126	GPR160	176	GPRC5B	226	MCHR2		
27	CALCRL	77	FPR2	127	GPR161	177	GPRC5C	227	MLNR		
28	CASR	78	FPR3	128	GPR162	178	GPRC5D	228	MRGPRD		
29	CCKAR	79	FSHR	129	GPR17	179	GPRC6A	229	MRGPRE		
30	CCKBR	80	GABBR1	130	GPR171	180	GRM1	230	MRGPRF		
31	CCR10	81	GAL1	131	GPR173	181	GRM2	231	MRGPRG		
32	CCR2	82	GAL2	132	GPR174	182	GRM4	232	MRGPRX1		
33	CCR3	83	GAL3	133	GPR18	183	GRM5	233	MRGPRX2		
34	CCR4	84	GCGR	134	GPR182	184	GRM6	234	MRGPRX3		
35	CCR5	85	GHRHR	135	GPR183	185	GRM7	235	MRGPRX4		
36	CCR6	86	GHSR	136	GPR19	186	GRM8	236	MTNR1A		
37	CCR7	87	GIPR	137	GPR20	187	GRPR	237	MTNR1B		
38	CCR8	88	GLP1R	138	GPR21	188	HCA1	238	NMBR		
39	CCRL2	89	GLP2R	139	GPR22	189	HCA2	239	NMUR1		
40	CD97	90	GNRHR	140	GPR25	190	HCA3	240	NMUR2		
41	CHRM1	91	GPBA	141	GPR26	191	HCTR1	241	NPBW1		
42	CHRM2	92	GPER	142	GPR27	192	HCTR2	242	NPBW2		
43	CHRM3	93	GPR1	143	GPR3	193	HRH1	243	NPFF1		
44	CHRM4	94	GPR101	144	GPR31	194	HRH2	244	NPFF2		
45	CHRM5	95	GPR110	145	GPR32	195	HRH3	245	NPS		
46	CMKLR1	96	GPR111	146	GPR34	196	HRH4	246	NPY1R		
47	CNR1	97	GPR113	147	GPR35	197	HTR1A	247	NPY2R		
48	CNR2	98	GPR114	148	GPR37	198	HTR1B	248	NPY4R		
49	CRHR1	99	GPR115	149	GPR37L1	199	HTR1D	249	NPY5R		
50	CRHR2	100	GPR116	150	GPR39	200	HTR1E	250	NTSR1		
